# Effect of Exercise on Regulating miRNA Expression in Brain Health and Diseases

**DOI:** 10.3390/biology14060729

**Published:** 2025-06-19

**Authors:** Jian Zhang, Fengmei Gu, Anand Thirupathi

**Affiliations:** 1 College of Physical Education, Chuzhou University, Chuzhou 239000, China; zhangjian_516@163.com; 2 Faculty of Sports Science, Ningbo University, Ningbo 315211, China; anand@nbu.edu.cn

**Keywords:** physical exercise, miRNA, neurodegenerative diseases, aerobic exercise, resistance exercise, brain

## Abstract

Physical exercise induces epigenetic modifications that significantly influence the expression of a set of small non-coding RNAs known as miRNAs. These changes can enhance exercise performance and impact the neurological system, suggesting that understanding miRNA-mediated mechanisms during exercise may offer valuable insights into using exercise as a therapeutic approach for neurodegenerative diseases. The existing literature on exercise-induced molecular pathways often presents inherent biases, complicating the establishment of exercise-induced miRNAs as reliable biomarkers for various chronic conditions due to their variable expression at tissue, organ, and systemic levels. Specifically, miRNA expression can downregulate critical signaling pathways such as TGF-β, FOXO, and NOTCH, which are implicated in the progression of neurodegenerative diseases. Exercise can override this scenario by targeting the expression of these miRNAs. However, the link between exercise modality-induced benefits (types, intensity, and duration) and the miRNA expression in brain cells is poorly understood. Therefore, this review aims to discuss how exercise-mediated miRNA expression affects brain pathophysiology, particularly in the context of neurodegenerative diseases.

## Simple Summary

Since miRNAs are fundamental players in regulating gene expression, understanding their involvement in various biological processes could offer therapeutic possibilities for chronic conditions, including cardiovascular diseases and neurological disorders. However, their expression patterns in various tissues, especially under diseased conditions, are challenging to establish, as single miRNAs target multiple genes, which may either exacerbate or mitigate the symptoms of these chronic conditions. Additionally, establishing miRNA-based therapeutics in the complex brain system is challenging, as this can induce off-target effects that may lead to undesirable side effects. Therefore, introducing physical exercise as a complementary therapy for enhancing miRNA-mediated benefits could help overcome these challenges. Hence, this review highlights the relevance of physical exercise-mediated miRNA benefits in brain health and diseases.

## 1. Introduction

The interplay between physical exercise, miRNA, and brain physiopathology is a rapidly evolving field. This was initiated by the groundbreaking discovery of *lin-4* in 1993 by Victor Ambros and his colleagues, who established the concept that a small subset of non-coding RNA, known as microRNA (miR), regulates nearly 30% of biological processes within the entire genome by post-transcriptionally modifying various genes [1]. Simultaneously, how this *lin-4* pleiotropically regulates another gene, called *lin-14,* could support the idea that these miRNAs significantly alter complex cellular processes in a pleiotropic manner [2]. Therefore, understanding the biogenesis of miRNAs may reveal the mechanism by which these miRNAs mediate their functions in brain cells. miRNA biogenesis begins with the action of RNA polymerase II (Pol II) in the nucleus that transcribes these miRNA genes into pri-miRNAs for capping, polyadenylation, and splicing to form a “hairpin” structure, which is then exported into the cytoplasm where Dicer cleaves pre-mature into mature ~22-nucleotide-long miRNA duplexes [3,4]. Then, they can post-transcriptionally modify the expression of the targeting genes by binding to the 3′ untranslated region (3′UTR) of their target genes, and rarely bind to the 5′UTR or the open reading frame of their target genes [5]. However, the evidence of miRNAs regulating target genes associated with various cellular processes in different diseases is often misleading. In addition, an overwhelming number of miRNAs (more than 40 million) and their interactions with targeting genes are difficult to establish specifically in the brain cells, which have limited regenerative capacity and sensitivity, and a blood–brain barrier (BBB) [5]. Therefore, establishing the factors that influence the expression of miRNAs, such as c-Myc, p53, and E2F, could aid in targeting specific genes [1,2,3]. Physical exercise is one of the non-invasive factors that affect the expression of miRNAs by regulating c-Myc, p53, and E2F [6]. For example, c-Myc directly controls the expression of the miR-17-92 cluster and miR-34a, and acute resistance exercise increases c-Myc expression, which leads to an increase in the expression of miR-17-92 and miR-34a [6,7], indicating the role of physical exercise in influencing miRNA expression and promoting miRNA-mediated benefits from tissue-specific to organ-specific or even systemic levels [3,8]. Additionally, the exercise-induced expression of miRNAs plays a crucial role in promoting beneficial adaptations during exercise, enhancing metabolism, cardiovascular health, muscle remodeling, and reducing inflammation. All of these could help to increase exercise performance. For example, Poy et al. reported that tissue-specific miRNAs, such as miR-375, regulate insulin secretion to trigger glucose-induced biological response [9], which enables adaptation to higher metabolic demands during exercise, thereby improving exercise performance [9]. Nevertheless, each exercise protocol (type and duration) induces different responses to miRNA activation, making it challenging to prescribe exercise as a primary tool for achieving miRNA-mediated benefits to improve the clinical outcomes of neurodegenerative diseases. For instance, a study reported that miR-375 decreases insulin gene expression by targeting myotrophin [10], while other miRNAs, including miR-124 and let-7b, can also target the same protein for metabolic regulation [11,12]. However, the role of specific exercise protocols in selectively targeting these miRNAs is unknown, particularly in the context of neurodegenerative diseases.

Establishing the link between exercise and miRNA expression in humans began with two studies in 2007 that reported that chronic exercise with a single bout altered the specific miRNA levels in the blood and muscles called muscle-specific miRNAs (myomiRs) [13,14], which indirectly paved the way for understanding myomiRs’ responses to exercise. Since then, several studies have investigated the effects of various exercise protocols on a broad range of miRNAs across various tissues and biofluids [15,16,17,18]. For example, aerobic exercise is linked to altering the miRNA expression profiles, such as miR-46a, miR-21, and miR-133, to regulate metabolism and cardiovascular health [15], while resistance training tends to enhance miR-1, miR-23a, miR-133a, and miR-133b, which are linked to muscle growth and repair [16]. In addition, high-intensity exercises increase miR-133a and miR-133b to improve the whole body metabolic profile and immediate adaptation [17], while long-term aerobic training may result in sustained changes in miRNA expression, like miRNA-223, to reduce the occurrence of atherothrombotic events and improve the chronic health benefits [18]. Furthermore, circulating miRNAs can serve as useful biomarkers for monitoring exercise adaptation and recovery. Although the ability of exercise to modulate miRNA expression in multiple tissues highlights its role as a systemic regulator of physiological processes, the lack of molecular mechanisms induced by miRNA that carry a tissue-specific response to a systemic level may require further research, especially to understand the role of altered miRNA in the exercised brain. Therefore, this review aims to discuss how exercise affects miRNA expression in the context of improving outcomes of neurodegenerative diseases.

## 2. How Does Exercise Affect the Biogenesis of miRNAs?

**Figure 1:**
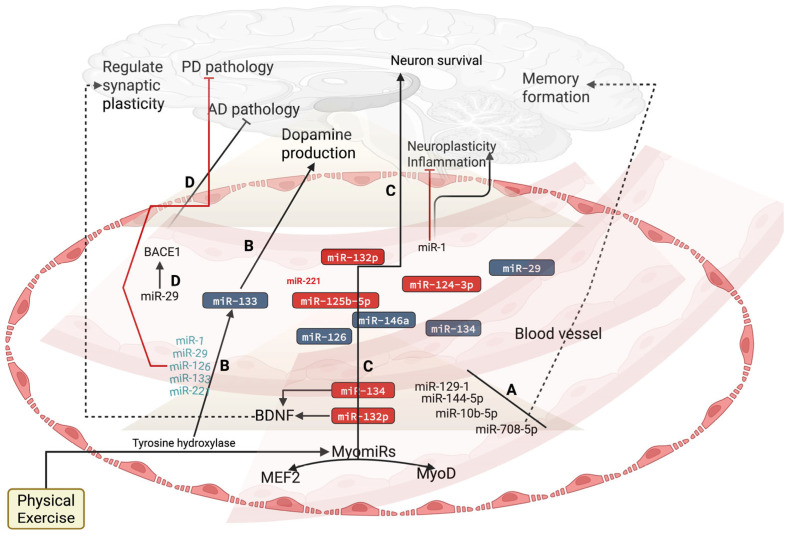
Physical exercise-induced myomiRs on brain health. (A) Exercise-triggered MyoD1 and MEF2 increase the release of myomiRs, such as miR-129-1-3p, miR-144-5p, and miR-10b-5p, from the muscle, which then enter the circulation to improve brain development by controlling neuronal growth and survival genes. Dotted lines indicate that miR-708-5p improves memory formation. (CB) Exercise-induced tyrosine hydroxylase expression enhances miR-133 in both muscle and circulation by targeting Ptx1, thereby improving memory formation and dopamine production. Exercise-induced muscle miR-134, miR-132p, miR-125b-5p, miR-126, miR-146a, miR-133a, and miR-206 increased the binding of BDNF/Trf to activate MAPK and ERK. This can enhance the BDNF-mediated benefits, including reduced neural inflammation, improved synaptic plasticity, and enhanced neuronal survival. However, overtraining decreases miR-34a, reducing the BDNF-induced benefits. (D) Exercise-induced miR-29 activates BACE1, thereby enhancing neuronal maturation, improving neuronal communication, and promoting neurogenesis.

It is known that Pol II establishes the transcription of miRNAs from small gene sequences. Recent evidence suggests that acute treadmill running upregulates muscle-specific Pol II to transcribe miR-451 during the transcriptional activity of PGC-1α [19], indicating the involvement of exercise in regulating miRNA expression by enhancing Pol II activity [19]. Additionally, a single bout of exercise increases histone acetylation [20]. This can increase Pol II recruitment to influence miRNA expression [21]. However, no studies in the literature have established the influence of exercise on Pol II in the brain, especially in neurodegenerative diseases, but studies have established the role of exercise in regulating miRNAs, such as miRNA-29, miR-132, miR-133, miR-129-1-3p, miR-144-5p, miR-10b-5p, and miR-708-5p, to improve neuronal maturation, neuronal communication, and neurogenesis by targeting beta-secretase 1 (BACE1), brain-derived neurotrophic factor (BDNF), and tyrosine hydroxylase through a transcription factor, paired-like homeodomain 1 (Ptx1), for dopamine production and memory formation, without assessing Pol II (Figure 1) [22,23,24]. Additionally, miRNA biogenesis components, such as Drosha, Dicer, and Exportin-5, are enhanced by exercise. For example, 3 h of moderate-intensity cycling exercise increased the activity of Drosha, Dicer, and Exportin-5 [11,14,19,25]. This can upregulate the transcriptional activity of miRNAs. Furthermore, the exercise-induced activation of myoblast determination protein 1 (MyoD1) and myocyte enhancer factor 2 (MEF2) increased the release of myomiRs, such as miR-129-1-3p, miR-144-5p, miR-10b-5p, and miR-708-5p, and all these myomiRs are crucial in brain development by controlling the expression of genes related to neuronal growth and survival [14,23]. For example, the exercise-induced activation of MEF2 regulates neuronal development and synaptic plasticity, thereby enhancing learning and memory formation through these miRNAs [24,25,26]. A recent study has demonstrated that MyoD exhibits pro-neuronal activity, which reprograms neurons by shutting down the muscle program via Myt1l [27]. Moreover, RNA/ADAR editing patterns can influence miRNA biogenesis and function. Exercise alters the RNA/ADAR editing patterns in PD to influence miRNA biogenesis and function [28,29]. For example, resistance rehabilitative training for 16 weeks in PD patients improved the RNA/ADAR editing patterns [28]. RNA methylation, such as N6-methyladenosine (m6A), modifies miRNA biosynthesis, and endurance exercise decreases the m6A level [22,30]. Other regulatory mechanisms, such as argonaute and adenylation, also modify miRNA biosynthesis [31], and exercise regulates these processes to enhance miRNA synthesis [32,33,34].

## 3. Effect of Exercise on Circulatory miRNAs in Brain Health

Exercise effects are not tissue-specific. So, the systemic effect of exercise influences the circulatory miRNAs. For example, exercise-induced miRNAs are released within exosomes, and exercise increases the circulation of these miRNA-loaded exosomes to enhance systemic adaptation [35], evidenced by the short-cycling exercise that increases miRNAs, such as miR-1, miR-208a, and miR-33a, which are found in exosomes [36]. Additionally, miRNAs are regulated by high-density lipoprotein (HDL) [37]. Studies have shown that HDL-bound miRNAs, specifically miR-223-3p and miR-181c-5p, enhance angiogenesis and reduce vascular-related complications in a mouse model [26,37]. In this case, exercise could be an effective tool to improve HDL levels, thereby enhancing the protein binding characteristics of HDL, which facilitates effective miRNA transportation into the cytoplasm [37]. In particular, HDL-bound miRNAs are more stable using scavenger Receptor class B type I (SR-BI), rather than using endosomal/lysosomal pathways [38]. This can improve the functional integrity of miRNAs [38]. For example, HDL-bound miR-223 prevents atherosclerosis by regulating cholesterol metabolism [39]. HDL-bound miR-223 can also suppress the expression of intercellular adhesion molecule 1 (ICAM-1) and pro-inflammatory genes, thereby decreasing neuroinflammation [40]. miRNAs, such as miR-758, miR-144, and miR-302a, regulate the HDL-C metabolism [39]. However, the mechanism by which exercise facilitates the transportation of miRNA across the BBB by regulating tight junctions, efflux transporters, and enzymatic degradation to enter the BBB remains to be investigated. Additionally, a lack of specific transporters for miRNAs may potentially inhibit their entry into the BBB [41]. Exercise-induced muscle secretion, including myomiRs (such as miR-1, miR-133a, miR-133b, and miR-499) and exosomes, may have the ability to circumvent the BBB. This enhances the communication interface between muscle and neurons, thereby supporting the muscle–brain axis [42]. Such mechanisms can alter the genetic profile of targeted cells, potentially improving treatment strategies for neurodegenerative diseases [42]. For example, exercise-induced miRNAs, such as miR-125b-5p, miR-126, and miR-146a, target the BDNF gene, including the Val66Met polymorphism, which is linked to major depressive disorder (MDD) with Alzheimer’s disease (AD) [43], by improving synaptic remodeling and differentiation via BDNF/TrkB binding in MDD and AD by activating its downstream signaling, such as MAPK/ERK [44]. Other circulatory miRNAs, such as 125 b-5p, miR-126, and 146a, also fluctuated by acute exercise, and all of them are significantly upregulated in AD [45,46]. Therefore, characterizing these miRNAs during exercise could help diagnose the early onset of several neurodegenerative conditions. In addition, miRNAs such as miR-1, miR-29, miR-126, miR-133, and miR-221 are often downregulated in Parkinson’s disease (PD) [47]. Exercise, particularly aerobic training, modulates PD-related gene expression by targeting these miRNAs [46].

## 4. Exercise-Mediated Molecular Signaling on miRNA Expression to Reverse Brain Physiopathology

### 4.1. Aerobic Exercise-Mediated Molecular Signaling on miRNA Expression

**Figure 2:**
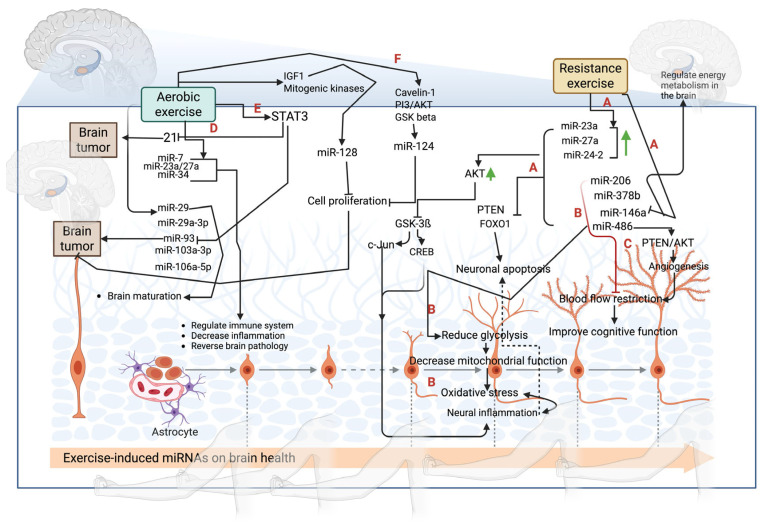
Exercise-induced miRNAs regulate the immune system, decrease inflammation, and reverse brain pathology. (A) Resistance exercise activates miRs-23/27a/24-2, which in turn activate AKT signaling. This can decrease neural inflammation by inhibiting GSK-3beta/c-Jun/CREB signaling and decrease amyloid formation. Also, these miRNAs inhibit PTEN and FOXO1 signaling to reverse neuronal apoptosis. (B) Exercise-regulated miRs-206/378b/146a improve energy metabolism and reduce oxidative damage in the hypoxia-mediated injuries in the brain by targeting Traf6 and Nf-kb. (C) Exercise-induced miR-486 and miR-221 activate PTEN/AKT signaling to improve cognition through modulating endothelial function and decrease cerebral ischemia. (D) Aerobic exercise activates miRs-7/23a/27a/34 to improve immune function. (E) Aerobic exercise activates STAT3 to inhibit miRs-21/93 and reduce brain tumors. (F) Additionally, aerobic exercise activates miR-124 by targeting caveolin-1, PI3K/AKT, and GSK-β. This can reduce cell proliferation in the tumor. (Green arrows indicate the increase of miRs, such as miRs-23/27a/24-2 and AKT phosphorylation).

Exercise-mediated signaling improves the expression of miRNAs and vice versa. However, different forms of physical activity uniquely modulate molecular pathways through targeting specific miRNA. For example, running wheel exercise reduced the expression of signal transducer and activator of transcription 1 and 3 (STAT1 and STAT3) and toll-like receptor (TLR) signaling pathways, thereby improving cognition in mice [48]. This cognitive benefit is linked to the upregulation of miR-181b, as reported by Chen et al. (2020) [49]. In contrast, treadmill exercise improves the JAK–STAT, NOD-like receptor, and Wnt pathways, while dampening calcium signaling, to enhance cognition in AD mice, and improve the neural function recovery in spinal cord injury in rats [50,51], primarily through the targeting of miR-21 (Figure 2) [52]. Swimming exercise-induced MiR-128 improves mitochondrial homeostasis via the miR-128/IGF-1 signaling pathway [52] and targets mitogenic kinases [53,54]. Despite the limited exploration of miR-128′s role concerning exercise and brain function, Shvarts-Serebro et al. demonstrated that running wheel exercise can also modulate miR-128, ultimately enhancing synaptic properties in the hippocampal neurons of AD mice [55]. Furthermore, miR-124 has been implicated in medulloblastoma development by targeting the solute carrier family 16, member 1 [56]. The wheel-running training program regulates miR-124 to inhibit age-related cognitive decline by modulating the signaling pathways of caveolin-1, phosphoinositide 3-kinase/protein kinase B (PI3K/Akt), and glycogen synthase kinase-3β (GSK-3β) [57]. Notably, exercise preconditioning achieved through a 30 min running program over three consecutive days has been shown to elevate circulating exosomal levels of miR-124 while minimizing apoptosis in rats with cerebral ischemia–reperfusion injury. The underlying mechanism appears to involve the modulation of the STAT3 and BCL-2/BAX signaling pathways [58]. Additionally, a voluntary running program for 4 weeks in the senescence-accelerated SAMP8 mouse suppresses miR-132, thereby improving cognition [59]. This effect may be mediated, in part, by the inhibition of inducible nitric oxide synthase (iNOS) expression through mitogen-activated protein kinase (MAPK) in the hippocampal region [60]. Moreover, the weighted wheel running training protocol alters miR-29 to downregulate BACE1, thereby preventing the accumulation of Aβ plaques and slowing AD progression by reducing the Dicer gene [61]. This regulatory mechanism occurs by targeting nuclear factor-kappa B (NF-κB) signaling pathways [62]. The reduction in miR-7 has also been linked to improvements in mitochondrial functionality and antioxidant capacity observed in master athletes [63]. A study reported that miR-7 is essential for reducing brain damage and enhancing motor recovery by repressing α-synuclein [64]. Exercise like half-marathon running increases miR-1, miR-133a, and miR-206 to modulate energy metabolism and intracellular Ca^2+^ levels, regulate synaptic activity, improve memory and learning, and enhance control proliferation in the brain by influencing insulin-like growth factor 1, (IGF-1), PI3K, sarcoplasmic/endoplasmic reticulum Ca2+-ATPase (SERCA-2), ras homolog enriched in brain (RHEB), synaptic Ras GTPase-activating protein 1 (RAS GAP1), and cyclin-dependent kinase 9 (CDK9) signaling [65,66,67]. Cycling exercise for 3 days at 70% VO_2max_ for 60 min decreased miR-486 in the circulation [68]. This can promote angiogenesis following cerebral ischemic injury by regulating the PTEN and Akt pathways [67]. Running a half-marathon increased the levels of miR-1, miR-133a, and miR-206 [69,70], and miR-133a and miR-206 are implicated in the exacerbation of cerebral ischemia/reperfusion injury and the pathogenesis of depression by targeting the BDNF pathway [71,72]. Cycling ergometer exercise for an 8-week period (30 mins three times a week) improved cognitive function in individuals with PD by increasing the levels of miR-106a-5p, miR-103a-3p, and miR-29a-3p [73].

### 4.2. Resistance Exercise-Mediated Molecular Signaling on miRNA Expression

Placing a high mechanical load on the muscle during resistance training induces the expression of several miRNAs [74]. For example, functional overload regulates the expression of miR-1 and miR-133, which in turn activate the IGF/AKT signaling pathway [74]. This scenario increases the translocation of neuronal GLUT4 from intracellular pools to nerve membranes [75]. This can improve the hippocampal memory process [76]. High-intensity resistance training increases the expression of miR-23a, miR-27a, and miR-24-2, which enhances muscle mass by restoring protein kinase B (AKT) signaling and decreasing the expression of phosphatase and tensin homolog (PTEN) and forkhead box protein O1 (FOXO1), thereby reducing inflammation [77]. The increase in FOXO1 is responsible for AD pathogenesis, mediated by the signaling of FOXO pathways via the PI3K/AKT and JNK/c-Jun pathways [78]. High-intensity resistance training can also alter the levels of miR-133a, miR-378b, miR-146a, and miR-486, all of which are associated with neurological disease, by targeting the PTEN, tuberous sclerosis complex-2 (TSC-2), and FOXO signaling pathways [69,79]. For example, miR-133a and miR-378b are involved in cerebral ischemia/reperfusion injury [71,72], and the dysregulation of miR-146a increases cerebrovascular disease, neurodegenerative diseases, and neurological tumors [80,81] by increasing neuroinflammation and oxidative stress, and reducing glycolysis and mitochondrial functions in glial cells [82]. Resistance exercise enhances glycolytic metabolism by targeting miR-146, thereby improving energy homeostasis in glial cells [82]. Moreover, high-intensity resistance training decreases miR-206 after 2 h of acute training, which may increase blood flow restriction to improve cognitive function [83,84].

### 4.3. Effect of Different Exercise Intensities on miRNA Expression

Exercise intensity plays a significant role in the expression of miRNA across various types of exercise, with each type influencing miRNA expression differently, even within the same endurance category. For instance, moderate-intensity running during a marathon leads to an increase in circulating miRNAs. In contrast, high-intensity swimming results in the differential expression of specific miRNAs, such as the miR-200 family, in the rat’s brain by targeting BDNF, Igf-1, and VGF [85]. DA Silva ND et al. reported that high-volume swimming exercise in the ninth and tenth weeks enhanced the expression of miR-126, promoting angiogenesis [86]. Additionally, combined training, which includes resistance training and high-intensity interval training (HIIT), may have a more pronounced effect on miRNA expression compared to HIIT alone. For example, Telles et al. demonstrated that resistance exercise and concurrent training increased the expression of miR-23a-3p and miR-206 in skeletal muscle, surpassing the results seen in those engaging in HIIT [87]. Overtraining can also impact miRNA expression. For example, Xu et al. indicated that 8 weeks of overtraining with treadmill exercise reduced the BDNF response by targeting miR-34a in the hippocampus of mice [88]. Furthermore, the extension of miRNA expression may be associated with prolonged exercise duration. For instance, research has shown that both aerobic and resistance training at varying intensities failed to elevate miRNA expression, suggesting that achieving meaningful increases in miRNAs requires at least six months of uninterrupted exercise performance [89].

## 5. Exercise-Mediated miRNAs on Oxidative-Related Stress in Brain Pathophysiology

**Figure 3:**
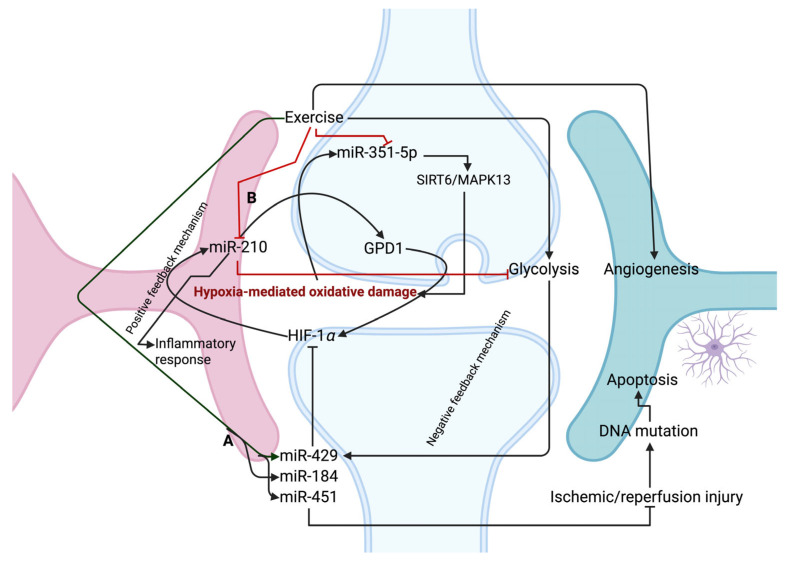
Exercise-induced miRNAs are involved in the oxidative damage process in the brain. (A) Exercise-activated miRNAs, such as miR-429/184/451, decrease ischemic/reperfusion injury by targeting oxidative-mediated damage. (B) miR-210 triggers HIF-1α via GPD1 to induce pro-inflammatory response caused by hypoxia. However, exercise decreases miR-210 to improve metabolic homeostasis by targeting HIF-alpha, while exercise-induced miR-429 inhibits HIF-alpha to decrease hypoxia-mediated oxidative damage via a negative feedback mechanism. Exercise regulates miR-351-5p to decrease endoplasmic reticulum stress via exerting anti-ferroptotic effect.

Due to high metabolic activities, the brain is more susceptible to oxidative damage. On the other hand, the accumulation of reactive oxygen species (ROS) increases the expression of miRNA involved in the process linked to oxidative damage [90]. For example, miR-351-5p increases oxidative injury by targeting sirtuin 6 and MAPK13, resulting in oxidative damage-mediated neural cell death [90,91]. This may be due to the interaction between oxidative stress and unbalanced mitochondrial fission, which leads to the death of neural progenitor cells in the hippocampus [90,91,92,93,94]. In contrast, miR-351-5p is also involved in reducing lipid peroxidation to alleviate endoplasmic reticulum stress by exerting anti-ferroptotic effects through the targeting of 5-lipoxygenase in traumatic brain injury [95]. Exercise regulates the expression of miR-351-5p to alleviate oxidative damage-mediated neural death in the hippocampus [96,97,98]. A recent study has shown that maternal exercise in pregestational diabetes normalizes the dysregulated miR-351-5p in female mice [99]. Oxidative damage and the hypoxia process are interlinked, and miRNAs presumably mediate this scenario. For instance, hypoxia can activate miR-210 through the interaction of hypoxia-inducible factor-1α (HIF-1α) [98]. On the other hand, miR-210 can induce HIF-1α through glycerol-3-phosphate dehydrogenase 1-like, which activates HIF-1α proline hydroxylation (Figure 3) [100]. Therefore, inhibiting miR-210 could suppress the pro-inflammatory response caused by hypoxia. A recent study reported that inhibiting miR-210 reduced acute brain injury by decreasing the pro-inflammatory response in ischemic stroke conditions [101]. Acute exercise decreased the level of miR-210 in response to hypoxia, thereby inhibiting apoptosis, promoting mitochondrial respiration over glycolysis, and enhancing angiogenesis [98]. Conversely, HIF-1α induces miR-429 expression, which decreases HIF-1α stability and regulates hypoxia through a negative feedback loop [102]. Exercise may increase miR-429 in response to glucose metabolism, thereby contributing to improved endothelial function and decreased hypoxia-mediated oxidative stress [103]. The oxidative modification of miR-184 can tone down the deleterious effects at the cellular and organismal levels [104]. For example, the inhibition of miR-184 increases glioma cell proliferation and invasion. Thus, it prevents the miR-184-mediated deletion of excess DNA mutations [105]. Resistance exercise upregulated miR-184, supporting the possibility that exercise increases miR-184 to mitigate DNA mutations in glioma cells [106]. ROS scavengers, including miR-451, can nullify oxidative stress-mediated apoptosis [107]. For instance, upregulated miR-451 protects neurons from ischemic/reperfusion injury by decreasing apoptosis [107]. Exercise training regulates miR-451, which enhances the oxidative capacity of mitochondria and their biogenesis (Table 1) [108]. Altogether, exercise-mediated miRNAs, such as miR-184, miR-210, miR-351-5p, and miR-429, are involved in regulating oxidative damage processes to decrease hypoxia-mediated pro-inflammatory responses in the brain.

## 6. Limitations of Using miRNAs as Drugs in Treating Brain Diseases

miRNA delivery across the BBB is challenging, as most miRNAs cannot efficiently cross the BBB; thus, brain cells fail to access them. In this case, the direct injection method, such as the use of viral vectors, can have additional drawbacks, including safety concerns in the brain. In addition, degrading enzymes like nuclease can degrade miRNAs before they reach the targeted brain cells, and the half-life of miRNAs in brain cells is shorter. In this case, exercise-mediated exosomes can help circulating miRNAs from RNase degradation. The pleiotropic effects of a single miRNA can target multiple genes, potentially causing unintended side effects in the brain. For example, miR-128 plays a crucial role in nervous system development by targeting Forkhead Box M1, cAMP response element-binding protein (CREB), splicing factor SC35, and LIM domain kinase 1 (Limk1) [138], and has also been linked to various types of brain cancer via targeting STAT5B and KRAS genes [139,140]. miR-9 targets the forkhead box protein G1 (FoxG1), hairy and enhancer of split-1 (Hes1) or homolog of the Drosophila tailless gene (TLX), CREB, and neurofibromin (NF1) genes for neuronal development [141], whereas the aberrant expression of miR-9 is linked with the glioma via targeting v-Myc avian myelocytomatosis viral oncogene homolog (Myc) and POU class 5 homeobox 1 (OCT4) in human glioma [142]. Furthermore, miRNAs trigger immunogenicity and toxicity in the brain. This is evidenced by the increase in several miRNAs, such as miR-10b, miR-221, miR-146a, and miR-146b, in various brain pathologies [143]. For example, miR-10b is implicated with the tumor cell growth and survival of glioma in human glioma cells by targeting phosphoglycerate kinase and insulin-like growth factor binding protein 2 [144]. The increase in plasma miR-133b and miR-221-3p is linked to early PD [145]. Nevertheless, translating preclinical findings into the clinical application of miRNA usage as drugs requires further exploration, as animal model results may not accurately reflect the complexity of human brain diseases. For example, miR-34a was implicated in neuronal loss and synaptic dysfunction in an AD mouse model [146], and the administration of anti-miR-34a oligonucleotides decreased neuroinflammation and improved cognitive functions in AD; obtaining these results mainly relies on post-mortem histological assays, and assessing the cognitive function of animal models is different from the neuropsychological testing used in humans, suggesting the careful consideration of these preclinical findings about miRNA-based drugs before they are translated into clinical application. Therefore, combining therapies like exercise with miRNA-based therapies could improve the outcomes of patients with brain diseases in various ways, such as improving drug delivery by influencing BBB permeability or blood flow, altering the target miRNAs, and improving downstream effects, and addressing the above-mentioned factors could unlock the full efficacy of miRNA-based drugs in treating a wide range of brain diseases.

## 7. Conclusions

In conclusion, while miRNAs hold promise as biomarkers for the early detection of brain pathology associated with neurodegenerative diseases, their specificity and expression patterns present challenges for their diagnostic application; however, exercise emerges as a beneficial intervention, capable of enhancing miRNA expression and functionality through multiple mechanisms. For example, exercise may significantly improve the effectiveness of miRNA as biomarkers by enhancing miRNA-inducing factors, such as c-Myc, p53, and E2F, promoting the circulation of miRNA-loaded exosomes, and modulating the underlying components of miRNA biogenesis, including Drosha and Dicer. Additionally, exercise-induced miRNAs released from various tissues (skeletal muscle, cardiac muscle, and adipose tissue), can cross the BBB to produce systemic effects in the brain, which could facilitate the development of evidence-based exercise programs for the prevention of neurological diseases. For instance, exercise-mediated skeletal muscle-derived circulatory miRNAs, such as miR-133a and miR-148b, have been linked to improving cognitive function, while cardiac muscle-derived circulatory miRNAs, including miR-17-3p, miR-133a, and miR-208a, may play a role in neuroprotective mechanisms. Furthermore, miRNAs (miR-21-5p, miR-93-5p, miR-155-5p, and miR-222-3p) from adipose tissue may influence inflammation in the brain, which is a contributing factor in conditions such as AD. Thus, incorporating regular physical activity could be a key strategy in advancing early diagnostic approaches for neurodegenerative diseases and enhancing overall brain health.

## Figures and Tables

**Table 1:** Effect of exercise-induced miRNAs on brain health.

miRNAs	Exercise Protocols	Possible Targeting Pathways	Effect of Exercise-Induced miRs on Brain Health	References
**miR-21 (** **↑** **)**	Running wheel exercise for 2 weeks	MAP2K3 and STAT3	Improves cognitive function, decreases cerebral edema, increases BBB permeability, and decreases STAT3 expression, reducing neuronal death	[109,110]
**miR-146-a (** **↑** **)**	Resistance training (warm up for 5 min, 30 min of circuit, and cool down 5 min).	NF-κB signaling	Reduces neuroinflammation by repressing NF-κB	[111,112]
**miR-221-3p (** **↑** **)**	Treadmill exercise	JAK, STAT, SNCA, and PARK2	Affects neural apoptosis and cell proliferation, and regulates autophagy and oxidative process	[113,114]
**miR-222 (** **↑** **)**	Two exercise protocols were performed. (1) Mice performed a forced swimming exercise for 4 weeks twice a day for 90 min for 7 days. (2) As voluntary exercise, mice performed a cage wheel exercise for 4 weeks.	ITGB8 and HMBOX1	Decreases inflammation and brain injury	[115,116]
**miR-133 (** **↑** **)**	Swimming exercise for 8 weeks and treadmill training for 8 weeks, 50 min, 23 m/min.	Tyrosine hydroxylase, HDAC4, MEFC2, and BDNF	Dopamine production, neuronal plasticity, and neurological recovery	[117,118]
**miR-129-1-3p (** **↑** **)**	4 weeks of treadmill running exercise	IGFBP-5 and *ITM2A*	Improves memory formation	[119]
**miR-144-5p (** **↑** **)**	4 weeks of treadmill running exercise	IGFBP-5 and *ITM2A*	Improves memory formation	[119]
**miR-10b-5p (** **↑** **)**	Resistance training at 70% of the 1RM	BDNF and HOX	Survival and differentiation of neurons	[120]
**miR-708-5p (** **↑** **)**	4 weeks of treadmill running exercise	BDNF, HOX, NNAT and SERCA	Perturbs calcium re-uptake into the ER and increases the leakage of calcium in the cytoplasm to induce bipolar disorder	[119,121]
**132-3p (** **↑** **)**	Running exercise for 10 km race	FOXO3, NFAT and HDAC3	Neuronal protection. Increases hippocampal cells	[122]
**124-3p (** **↑** **)**	Cognitive-exercise dual-task intervention	AMPK/mTOR pathway, caveolin-1 and PI3K/AKT and GSK3β pathways.	Inhibits neuronal apoptosis and increases neuronal development and cognitive functions	[123]
**miR-125b-5p (** **↑** **)**	Treadmill running exercise for 20 min with 80%	BDNF pathway and MMP-15	Improves cognitive dysfunction by inhibiting neuroinflammation	[124,125]
**miR-126 (** **↔** **)**	High-intensity running exercise at maximum speed for 4 × 30 s	Zonula occludens-1 and claudin-5 and occludin	Promotes angiogenesis and neurogenesis in cerebral ischemia	[126,127]
**146a (** **↑** **)**	Treadmill exercise for 5 days up to 60 min/day with 22 m/min speed for 60 days	IRAK1, TRAF6, and NF-kB	Decreases inflammation and apoptosis	[128,129]
**miR-221 (** **↔** **)**	Treadmill exercise for a total period of 4 weeks with a total speed of running 18 m/min.	PTEN/PI3K/pathway	Modulates endothelial function and decrease apoptosis in cerebral ischemia	[130]
**miR-128 (** **↑** **)**	Swimming exercise (a total period of 12 weeks, 5 days a week for 200 min)	IGF-1 signaling pathway and mitogenic kinases and PHF6	Regulates neuronal migration and neuronal development	[131]
**miR-93 (** **↑** **)**	8 weeks of HIIT as follows: 5 min warm-up; 5 min standard stretching at low intensity; then, 30 min running at an intensity of 75%	IL-1β, TNF, IL-6, TLR4, and STAT3	Axogenesis, inflammation, and metabolism of the brain	[132]
**miR-29a-3p (** **↑** **)**	Cycling ergometer exercise for 8-week period (30 min/3 times a week)	BACE1	Improves cognitive function	[73]
**miR-23a/27a and 34 (** **↑** **)**	Resistance training	AKT, PTEN, FOXO1, PI3, and JNK/C-Jun	Regulate immune system, inflammation, and amyloid formation	[77]
**miR-378b (** **↑** **)**	Acute resistance training as follows: leg press (50–70% of 1RM) for 45 min	ANRIL and ATG3	Decrease hypoxic–ischemic brain injury	[133]
**miR-210 (** **↑** **)**	Aerobic exercise (3 times per week, 8-week duration)	CDK10 and EFNA3	Improves angiogenesis and metabolism	[134]
**miR-486 (** **↓** **)**	Aerobic exercise for 60 min at 70% VO2 max	Decreases glutathione peroxidase 3 and Thioredoxin-like-1	Neurodegeneration	[135,136]
**miR-499-5p (** **↑** **)**	Treadmill exercise	Cav1.2	Regulates neuroplasticity	[137]
**miR- miR-451**	Swimming exercise	PGC-1α	Improves angiogenesis and decreases apoptosis	[108]

Note: (↑) increase; (↓) decrease; (↔) no change.

## Data Availability

Not applicable.

## Abbreviations

The following abbreviations are used in this manuscript:

MAP2K3	Dual specificity mitogen-activated protein kinase kinase 3
STAT3	Signal transducer and activator of transcription 3
NF-κB	Nuclear factor kappa-light-chain-enhancer of activated B cells
JAK	Janus kinase
PARK2	Parkin
ITGB8	Integrin beta-8
HMBOX1	Homeobox containing 1
HDAC4	Histone deacetylase 4
Mef2	Myocyte enhancer factor-2
BDNF	Brain-derived neurotrophic factor
IGFBP-5	Insulin-like growth factor-binding protein 5
ITM2A	Integral membrane protein 2A
NNAT	Neuronatin
SERCA	Sarcoplasmic/endoplasmic reticulum Ca^2+^-ATPase
AMPK	5′ AMP-activated protein kinase
mTOR	The mammalian target of rapamycin
GSK3β	Glycogen synthase kinase-3 beta,
MMP15	Matrix metalloproteinase 15
IRAK-1	Interleukin-1 receptor-associated kinase 1
TRAF6	TNF receptor associated factor
PTEN	Phosphatase and tensin homolog
IL-6	Interleukin 6
TLR4	Toll-like receptor 4
BACE1	Beta-secretase 1
ANRIL	Antisense non-coding RNA in the INK4 locus
Atg3	Autophagy related 3
EFNA3	Ephrin A3
Cav1.2	Calcium channel, voltage-dependent, L type, alpha 1C subunit
PGC-1α	Peroxisome proliferator-activated receptor gamma coactivator 1-alpha
Limk1	LIM domain kinase 1
CREB	cAMP response element-binding protein
NF1	Neurofibromin
